# Comparative Meta-Analysis: Salivary, Plasma, and Serum miRNA Profiles for Oral Squamous Cell Carcinoma Detection

**DOI:** 10.3390/jpm16010052

**Published:** 2026-01-16

**Authors:** Arbi Wijaya, Vera Julia, Nurtami Soedarsono, Turmidzi Fath, Bayu Brahma, Alif Rizqy Soeratman, Denni Joko Purwanto, Yutaro Higashi, Masaaki Miyakoshi, Tsuyoshi Sugiura

**Affiliations:** 1 Division of Oral Oncology and Surgical Sciences, Graduate School of Dentistry, Tohoku University, Sendai 908-0872, Miyagi, Japan; yutaro.higashi.b6@tohoku.ac.jp (Y.H.); masaaki.miyakoshi.e3@tohoku.ac.jp (M.M.); tsuyoshi.sugiura.b2@tohoku.ac.jp (T.S.); 2 Department of Oral and Maxillofacial Surgery, Faculty of Dentistry, Universitas Indonesia, Central Jakarta 10430, Jakarta, Indonesia; vera.julia@ui.ac.id; 3 Department of Oral Biology, Faculty of Dentistry, Universitas Indonesia, Central Jakarta 10430, Jakarta, Indonesia; nurtami@ui.ac.id (N.S.); turmidzi.fath02@ui.ac.id (T.F.); 4 Division of Surgical Oncology, Dharmais National Cancer Center Hospital, West Jakarta 11420, Jakarta, Indonesia; bbrahma@dharmais.co.id (B.B.);

**Keywords:** mirRNAs, saliva, serum, plasma, sensitivity, specificity

## Abstract

**Background:** MiRNAs have emerged as minimally invasive biomarkers with considerable potential for the early detection of oral squamous cell carcinoma (OSCC). Although numerous studies have evaluated circulating miRNAs across different biofluids, the comparative diagnostic performance of saliva-, serum-, and plasma-derived miRNAs has not been systematically clarified. **Methods:** A meta-analysis was performed by screening PubMed, MEDLINE, Scopus, CINAHL, and related databases. Nineteen eligible studies evaluating miRNA-based assays in saliva, serum, or plasma were included. A random-effects bivariate model was used to calculate pooled sensitivity, specificity, and area under the HSROC curve. Meta-regression using log diagnostic odds ratio (lnDOR) examined whether biofluid type significantly influenced diagnostic performance. **Results:** Salivary miRNAs showed a pooled sensitivity of 0.76 (95% CI: 0.68–0.82; I^2^ = 84.69%), specificity of 0.79 (95% CI: 0.70–0.85; I^2^ = 70.41%), and an AUC of 0.84 (95% CI: 0.80–0.87). Plasma miRNAs produced comparable results with a pooled sensitivity of 0.77 (95% CI: 0.61–0.88; I^2^ = 90.45%), specificity of 0.79 (95% CI: 0.63–0.89; I^2^ = 80.20%), and an AUC of 0.85 (95% CI: 0.81–0.89). Serum-derived miRNAs demonstrated the highest accuracy with a pooled sensitivity of 0.82 (95% CI: 0.70–0.90; I^2^ = 76.92%), specificity of 0.88 (95% CI: 0.75–0.95; I^2^ = 74.87%), and an AUC of 0.91 (95% CI: 0.89–0.94). Despite serum’s numerically superior performance, meta-regression revealed no significant matrix effect (Wald χ^2^ = 0.20, *p* = 0.903). **Conclusions:** Although serum-derived miRNAs performed best overall, biofluid type was not a statistically significant determinant of diagnostic performance.

## 1. Introduction

Oral cancer ranks as the sixteenth most common malignancy worldwide, with incidence and mortality strongly influenced by regional habits, ethnic predispositions, and environmental exposures [1]. In 2018, cancers of the lip and oral cavity accounted for 354,864 new cases and 177,384 deaths globally, and the overall five-year survival rate of newly diagnosed patients remains approximately 50%, reflecting the persistent global disease burden [1,2]. Among these malignancies, oral squamous cell carcinoma (OSCC) comprises nearly 90% of all cancers arising in the oral cavity, making it the dominant histological subtype of oral cancer [3].

The current diagnostic gold standard for OSCC is histopathological evaluation of tissue biopsy [4]. While highly accurate, biopsy-based diagnosis is inherently invasive, dependent on trained clinicians and experienced pathologists, and is limited by its inability to capture tumor heterogeneity, as an incisional biopsy represents only a small portion of a potentially heterogeneous lesion [4]. These limitations have strengthened the impetus to develop non-invasive molecular biomarkers that are useful for screening and early detection of the disease [5]. Nevertheless, no individual biomarker has yet been sufficiently validated for routine use in early OSCC detection or risk stratification, underscoring a significant unmet need in oral oncology [6].

Among the promising molecular candidates, miRNAs have emerged as highly relevant diagnostic biomarkers. MiRNAs are small non-coding RNAs of approximately 19–25 nucleotides that regulate gene expression at transcriptional and post-transcriptional levels through interactions with the 3′ untranslated region (UTR) of target mRNAs [7,8]. Dysregulated miRNA expression plays a pivotal role in tumor initiation, progression, invasion, and metastasis, acting either as oncogenes or tumor suppressors depending on the cellular context [9,10,11]. Furthermore, miRNAs exhibit tissue- and tumor-specific expression patterns, making them highly informative molecular signatures [12].

A major advantage of miRNAs is their exceptional stability in biofluids. They are protected from RNase activity through encapsulation within extracellular vesicles or association with protein–lipid complexes, enabling robust detection in saliva, plasma, and serum [8]. Multiple studies have confirmed the stability of circulating miRNAs even under challenging pre-analytical conditions [13,14,15,16,17]. Given these properties, miRNAs represent compelling candidates for liquid biopsy-based diagnostics, with different biofluids offering distinct biological and practical advantages. However, the results across studies remain inconsistent due to variations in detection platforms, normalization strategies, patient characteristics, and sample types. Importantly, the relative diagnostic performance across different biofluids remains unclear, and no consensus exists regarding which biofluid provides the highest accuracy for OSCC detection [18].

Given these uncertainties this meta-analysis aims to compare the diagnostic accuracy of salivary, plasma, and serum miRNA profiles to determine which biofluid provides the most reliable performance for OSCC detection.

## 2. Materials and Methods

### 2.1. Study Protocol

**Figure 1:**
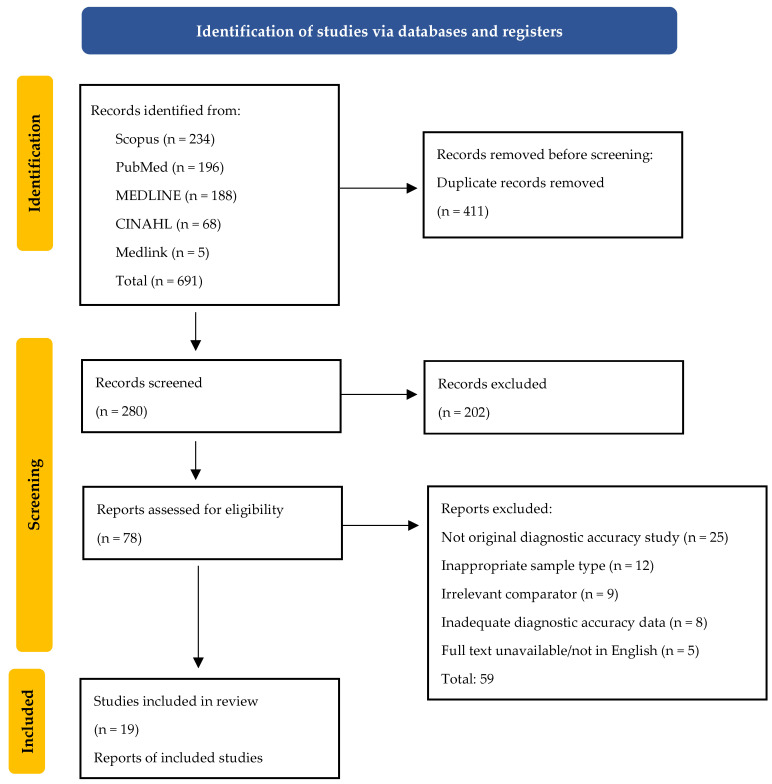
PRISMA (Preferred Reporting Items for Systematic Reviews and Meta-analysis) flow diagram of the study selection.

This meta-analysis was conducted in accordance with the Preferred Reporting Items for Systematic Reviews and Meta-Analyses (PRISMA) guidelines (Figure 1). All methodological steps including search design, eligibility screening, data extraction, and statistical synthesis were defined a priori to ensure transparency and reproducibility. The review protocol was prospectively registered in the International Prospective Register of Systematic Reviews (PROSPERO) under registration number CRD420251245619. The PRISMA checklist and flow diagram used in this study are presented in the Supplementary Material.

### 2.2. Search Strategy

A comprehensive systematic literature search was conducted by two independent reviewers (A.W. and N.S.) in the PubMed, MEDLINE, Scopus, CINAHL and Medlink databases to identify studies published between January 2015 and December 2025 that evaluated the diagnostic performance of microRNAs for detecting oral squamous cell carcinoma (OSCC). The search strategy incorporated MeSH terms and free-text keywords:

(“oral squamous cell carcinoma” OR OSCC OR “oral cancer”) AND (saliva OR salivary OR plasma OR serum) AND (microRNA OR miRNA OR “miR-21” OR “miR-31” OR “miR-184” OR “miR-200” OR “microRNAs”) AND (diagnos* OR sensitivity OR specificity OR ROC OR AUC OR “receiver operating”).

All records were imported into Rayyan “https://www.rayyan.ai/’ (accessed on 1 October 2025) for automated duplicate removal and blinded screening. Study selection proceeded through three predefined stages: database searching, title and abstract screening, and full-text evaluation according to the PICOS eligibility framework. Disagreements between reviewers (A.W. and N.S.) were resolved through discussion, and unresolved cases were adjudicated by a third reviewer (V.J.). The complete search process, including the number of studies identified, screened, excluded, and included, is illustrated in the PRISMA flow diagram.

### 2.3. Eligibility Criteria (PICOS Framework)

Study eligibility was defined according to the PICOS framework (Table 1). The Population consisted of patients with histopathologically confirmed oral squamous cell carcinoma (OSCC). The Intervention (Index Test) included the detection and quantification of microRNAs (miRNA/microRNA) in non-invasive biofluids, specifically saliva, plasma, or serum, using validated molecular platforms such as RT-qPCR, microarray, or next-generation sequencing. The Comparison group comprised healthy controls without any clinical evidence of disease. Eligible studies were required to report diagnostic accuracy outcomes, including sensitivity, specificity, area under curve (AUC), positive and negative predictive values (PPV, NPV), and sufficient data to derive true positives (TP), false positives (FP), true negatives (TN), and false negatives (FN). The Study design was restricted to observational diagnostic accuracy studies, including case–control and cohort designs. Studies were excluded if they did not assess diagnostic accuracy, involved inappropriate or non-specified biofluids, lacked extractable 2 × 2 contingency data, were non-original publications (such as reviews, letters, or editorials), were not available in English, or did not include a healthy control group.

### 2.4. Data Extraction

Two investigators (A.W. and N.S.) independently screened all eligible references by title and abstract, followed by full-text evaluation to extract relevant data according to a predefined protocol. The extracted information included: (1) study characteristics, encompassing study ID, year of publication, and country; (2) sample details, including total sample size and the type of biofluid analyzed (saliva, plasma, or serum); (3) miRNA-related features, such as the specific miRNAs evaluated, expression direction (upregulated or downregulated), whether the biomarkers were assessed individually or as part of a multi-miRNA panel, and the molecular detection platform used (RT-qPCR, microarray, or next-generation sequencing); and (4) diagnostic accuracy data, comprising true positives (TP), false positives (FP), true negatives (TN), false negatives (FN), sensitivity, specificity, positive predictive value (PPV), negative predictive value (NPV), and area under the ROC curve (AUC). All extracted data were cross-verified by both reviewers to ensure completeness and accuracy.

### 2.5. Quality Assessment

The methodological quality of each included study was independently evaluated by two examiners (A.W. and N.S.) using the Quality Assessment of Diagnostic Accuracy Studies-2 (QUADAS-2) tool. This instrument assesses four key domains: patient selection, index test, reference standard, and flow and timing to determine the risk of bias and, for the first three domains, concerns regarding applicability. Each domain was rated as low, high, or unclear risk based on predefined signaling questions, and a study was considered at high risk of bias if any signaling question within that domain indicated a potential methodological limitation. All assessments were conducted in duplicate, and any discrepancies or uncertainties were resolved through discussion. When consensus could not be reached, a third reviewer (V.J.) provided adjudication to ensure methodological rigor and consistency.

### 2.6. Statistical Analysis

All statistical analyses were performed using STATA version 19.0 (StataCorp, College Station, TX, USA). Pooled sensitivity, specificity, and AUC values were calculated using a random-effects bivariate model, with HSROC curves generated for overall and biofluid-specific (saliva, serum, plasma) analyses. Between-study heterogeneity was quantified using the I^2^ statistic, and potential contributors to heterogeneity were explored through meta-regression based on lnDOR. Funnel plots were used to assess possible publication bias across all studies and within subgroups.

## 3. Results

### 3.1. Study Characteristics

A total of 19 studies were included in this meta-analysis, comprising 1872 patients with OSCC and 783 healthy controls. The studies were conducted across multiple geographic regions, predominantly in Asia (China, Japan, Korea, India, Iran, Saudi Arabia, Turkey, and Taiwan; *n* = 13), followed by Europe (Hungary, Bulgaria, and Italy; *n* = 3), Australia (*n* = 2), and Africa/Middle East (Egypt; *n* = 1). The included studies evaluated a wide range of salivary, plasma, and serum microRNAs using RT-qPCR, microarray, or NGS platforms, assessing both single-miRNA markers and multi-miRNA panels. Detailed study characteristics including publication year, country, sample size, biofluid type, and miRNA features are presented in Table 2. Baseline demographic and clinical characteristics of OSCC patients and control groups across the included studies are summarized in Table 3, while comprehensive diagnostic accuracy data (TP, FP, TN, FN, sensitivity, specificity, PPV, NPV, and AUC) for each study are summarized in Table 4.

### 3.2. Data Analysis

We compared three diagnostic subsets based on biofluid type: saliva, serum, and plasma, and additionally performed a combined analysis integrating all miRNA matrices. A random-effects model was applied to pool sensitivity, specificity, and AUC estimates across all studies and within each biofluid-specific subgroup. Forest plots and HSROC curves were generated to illustrate overall diagnostic performance.

**Figure 2:**
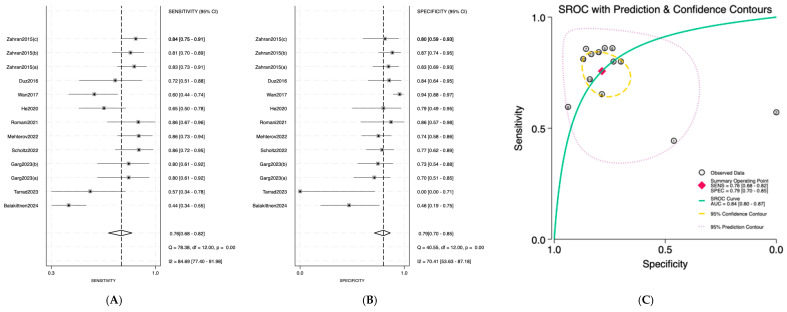
Diagnostic performance of salivary microRNAs for detecting OSCC. (**A**) Forest plot of pooled sensitivity of salivary microRNAs among 19 studies; (**B**) Forest plot of pooled sensitivity of salivary microRNAs; (**C**) SROC Curve with pooled estimates of sensitivity, specificity, and AUC of salivary microRNAs in OSCC. Abbreviations: AUC, area under the SROC curve; miRNA, microRNAs; OSCC, oral squamous cell carcinoma; SROC, summary receiver operator characteristic [19,20,21,22,23,24,25,26,27,28].

**Figure 3:**
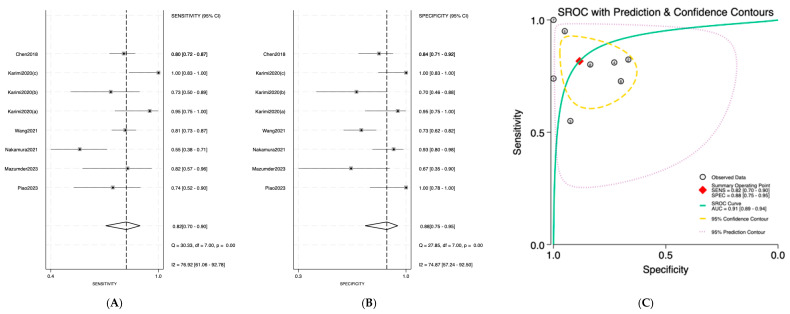
Diagnostic performance of serum microRNAs for detecting OSCC; (**A**) Forest plot of pooled sensitivity of serum microRNAs among 19 studies; (**B**) Forest plot of pooled sensitivity of serum microRNAs; (**C**) SROC Curve with pooled estimates of sensitivity, specificity, and AUC of serum microRNAs in OSCC. Abbreviations: AUC, area under the SROC curve; miRNA, microRNAs; OSCC, oral squamous cell carcinoma; SROC, summary receiver operator characteristic [29,30,31,32,33,34].

**Figure 4:**
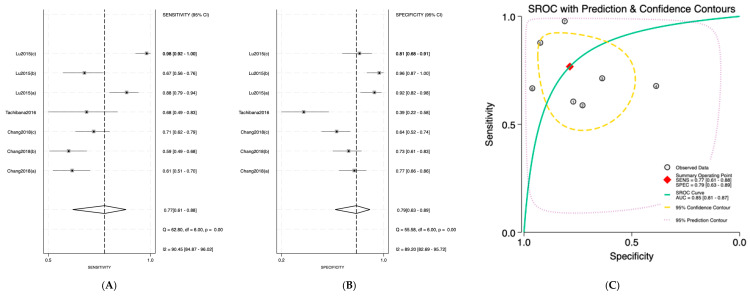
Diagnostic performance of plasma microRNAs for detecting OSCC. (**A**) Forest plot of pooled sensitivity of plasma microRNAs among 19 studies; (**B**) Forest plot of pooled sensitivity of plasma microRNAs; (**C**) SROC Curve with pooled estimates of sensitivity, specificity, and AUC of plasma microRNAs in OSCC. Abbreviations: AUC, area under the SROC curve; miRNA, microRNAs; OSCC, oral squamous cell carcinoma; SROC, summary receiver operator characteristic [35,36,37].

**Figure 5:**
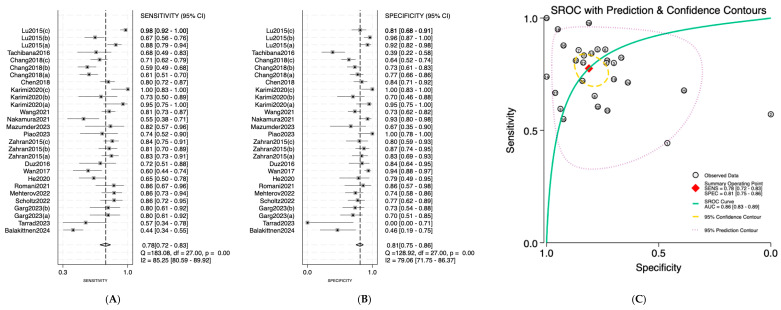
Diagnostic performance of combined sample (saliva, serum, and plasma) microRNAs for detecting OSCC. (**A**) Forest plot of pooled sensitivity of combined sample (saliva, serum, and plasma) microRNAs among 19 studies; (**B**) Forest plot of pooled sensitivity of combined sample (saliva, serum, and plasma) microRNAs; (**C**) SROC Curve with pooled estimates of sensitivity, specificity, and AUC of combined sample (saliva, serum, and plasma) microRNAs in OSCC. Abbreviations: AUC, area under the SROC curve; miRNA, microRNAs; OSCC, oral squamous cell carcinoma; SROC, summary receiver operator characteristic [19,20,21,22,23,24,25,26,27,28,29,30,31,32,33,34,35,36,37].

Salivary miRNAs demonstrated a pooled sensitivity of 0.76 (95% CI: 0.68–0.82; I^2^ = 84.69%), specificity of 0.79 (95% CI: 0.70–0.85; I^2^ = 70.41%), and an AUC of 0.84 (95% CI: 0.80–0.87) (Figure 2). Serum-derived miRNAs showed higher diagnostic accuracy, with a pooled sensitivity of 0.82 (95% CI: 0.70–0.90; I^2^ = 76.92%), specificity of 0.88 (95% CI: 0.75–0.95; I^2^ = 74.87%), and an AUC of 0.91 (95% CI: 0.89–0.94) (Figure 3). Plasma-based miRNAs yielded a pooled sensitivity of 0.77 (95% CI: 0.61–0.88; I^2^ = 90.45%), specificity of 0.79 (95% CI: 0.63–0.89; I^2^ = 89.20%), and an AUC of 0.85 (95% CI: 0.81–0.89) (Figure 4). When all three biofluids were combined, the overall pooled sensitivity was 0.78 (95% CI: 0.72–0.83; I^2^ = 85.25%), specificity was 0.81 (95% CI: 0.75–0.86; I^2^ = 79.06%), and the overall AUC was 0.86 (95% CI: 0.83–0.89) (Figure 5).

**Figure 6:**
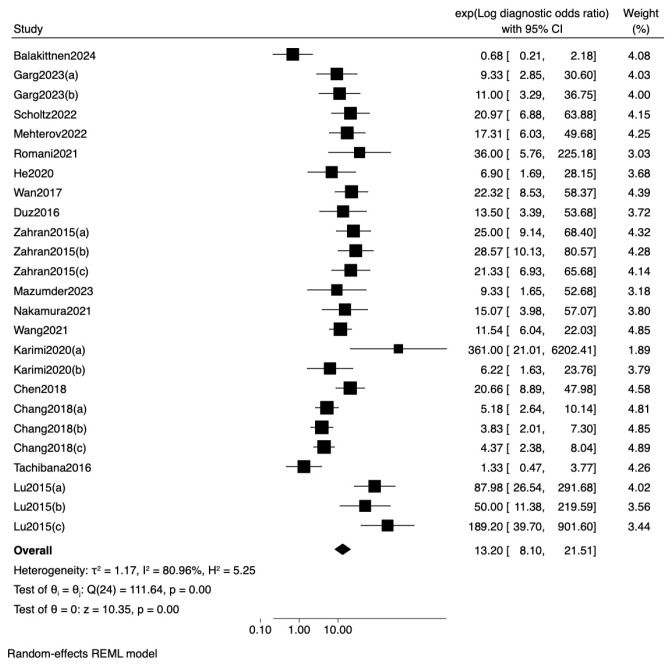
Forest plot and random-effects meta-regression of diagnostic odds ratios (DOR) for miRNA-based detection of oral squamous cell carcinoma (OSCC) across different sample [19,21,22,23,24,25,26,27,28,30,31,32,33,34,35,36,37].

To further examine the influence of sample type on diagnostic accuracy, a meta-regression analysis using log diagnostic odds ratio (lnDOR) as the effect size was conducted (Table 5). The model showed no statistically significant differences across matrices (Wald χ^2^ = 0.20, *p* = 0.9038). Compared with saliva (reference), neither serum (exp[b] = 1.38, *p* = 0.653) nor plasma (exp[b] = 1.13, *p* = 0.836) produced a meaningful change in DOR (Figure 6). Collectively, these findings indicate that the choice of biofluid does not materially modify the diagnostic performance of miRNA-based assays for OSCC.

**Figure 7:**
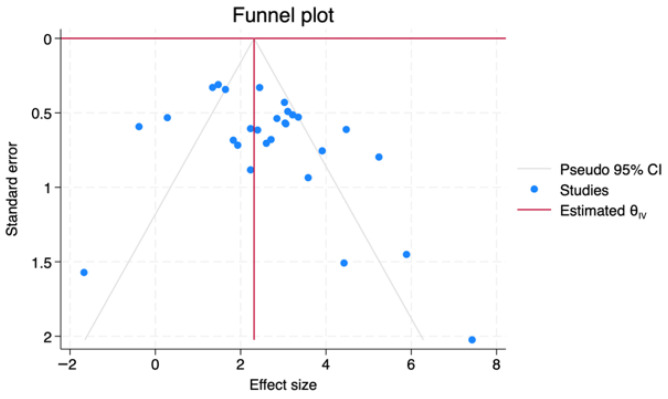
Funnel plots assessing potential publication bias for lnDOR across biofluid types.

Funnel plots were constructed by plotting the log diagnostic odds ratio (lnDOR) for each study against its standard error, allowing visual detection of small-study effects or publication bias (Figure 7). The saliva subgroup showed a relatively symmetric distribution, with studies clustering around the pooled lnDOR and narrowing at higher precision levels. Serum studies displayed wider scatter, particularly among lower-precision datasets, but without directional skew. Plasma studies, being fewer in number, produced a less distinct funnel shape, limiting interpretability due to small-study variance. The combined dataset demonstrated the most stable funnel structure, indicating no strong evidence of publication bias. Overall, visual inspection across all subgroups did not identify systematic asymmetry, supporting the assumption of unbiased reporting.

### 3.3. Result of Quality Assessment

**Figure 8:**
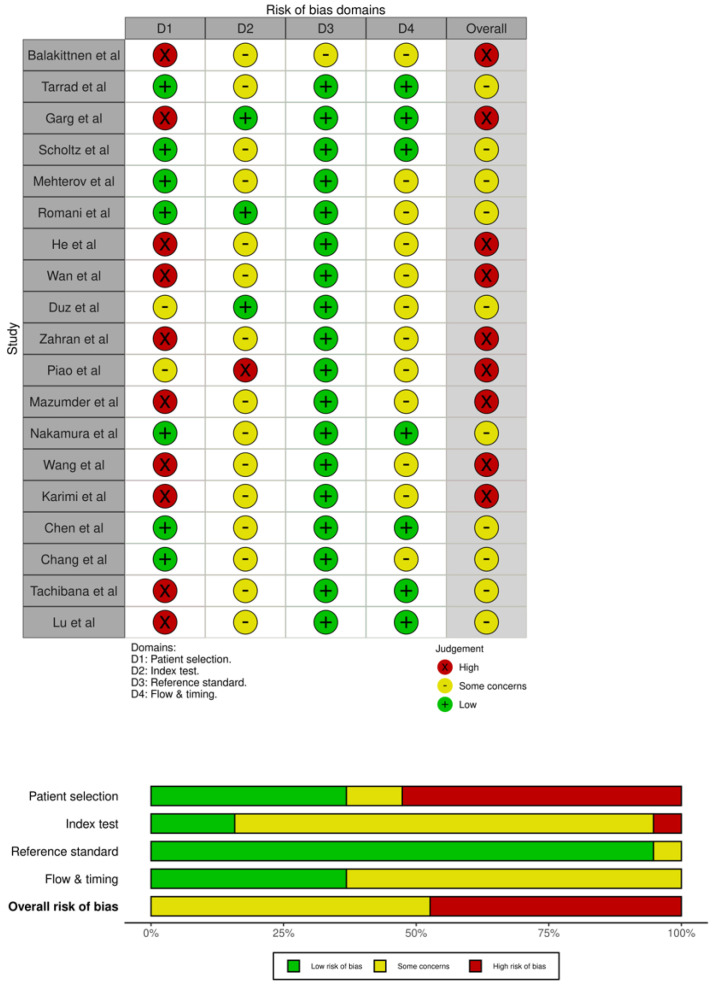
QUADAS−2 quality assessment of the 19 included articles. Abbreviations: QUADAS-2, Quality Assessment of Diagnostic Accuracy Studies [19,20,21,22,23,24,25,26,27,28,29,30,31,32,33,34,35,36,37].

The QUADAS-2 evaluation is summarized in Figure 8 Most studies demonstrated low risk of bias in the index test, reference standard, and flow/timing domains. However, patient selection frequently showed high or unclear risk, largely due to case–control designs and non-random sampling approaches. Applicability concerns were generally low across all domains. Overall, the methodological quality of the included studies was acceptable, with no critical limitations likely to substantially affect the pooled diagnostic estimates.

## 4. Discussion

In 2020, an estimated 377,713 new OSCC cases were reported worldwide [38]. Projections from the Global Cancer Observatory indicate that the incidence of OSCC will increase by nearly 40% by 2040, accompanied by a corresponding rise in mortality [39]. While early-stage OSCC (stages I and II) shows favorable five-year survival rates exceeding 80%, prognosis declines sharply to around 60% in advanced stages (III and IV) [31]. This disparity highlights the critical importance of early diagnosis. MiRNAs are now widely investigated as noninvasive diagnostic biomarkers for early diagnosis of OSCC [40,41].

In this meta-analysis, 19 studies evaluating miRNA-based diagnostic assays for OSCC were included across three biofluids: saliva, serum, and plasma. Collectively, a total of 54 unique miRNAs were identified. Of these, 38 were reported as upregulated in OSCC, 12 were downregulated, and four exhibited mixed or inconsistent expression patterns across studies. Among all investigated biomarkers, miR-21 emerged as the most frequently studied miRNA, appearing in three independent studies (Garg et al. [21], Zahran et al. [28], and Karimi et al. [33]). This finding highlights the biological relevance of miR-21, a well-known oncogenic microRNA (“oncomiR”) that promotes tumor proliferation, invasion, and metastasis through the suppression of multiple tumor-suppressor pathways especially in oral cancer [42]. The importance of miR-21 as a circulating biomarker has also been emphasized in a meta-analysis by Peng et al. that identified miR-21 as one of the most representative and extensively validated miRNAs across various cancer types, including colorectal cancer [43].

Building upon this biomarker profile, we next evaluated the pooled diagnostic performance across saliva, serum, and plasma. The pooled diagnostic estimates demonstrated notable variation among biofluid types. Salivary miRNAs yielded a pooled sensitivity of 0.76, specificity of 0.79, and an AUC of 0.84, indicating moderate diagnostic accuracy. Serum-derived microRNAs showed the highest performance, with a pooled sensitivity of 0.82, specificity of 0.88, and an AUC of 0.91, surpassing both saliva and plasma. Plasma microRNAs exhibited a pooled sensitivity of 0.77, specificity of 0.79, and an AUC of 0.85, comparable to saliva but still inferior to serum. When all matrices were combined, the overall pooled values were a sensitivity of 0.78, specificity of 0.81, and an AUC of 0.86. Among the three biofluids, serum consistently demonstrated superior diagnostic accuracy, achieving the highest sensitivity, specificity, and AUC, exceeding even the combined analysis of all biofluid types.

The superior diagnostic performance observed for serum-derived miRNAs in this meta-analysis likely reflects both biological and pre-analytical effects related to coagulation process [44,45,46,47]. Regarding the effects of the coagulation process, studies comparing serum and plasma collected simultaneously from the same individuals have consistently demonstrated higher total RNA and miRNA concentrations in serum, suggesting the release of additional miRNAs during coagulation [46]. Furthermore, a study reported significant differences in approximately one-third of measured miRNAs between serum and plasma, with higher miRNA levels observed in serum [47]. These differences were attributed to the serum preparation process, particularly clot formation, during which miRNA-containing blood cells may become incorporated into the clot and release additional miRNAs [47]. Differences in miRNA abundance have also been shown to correlate more closely with platelet and leukocyte-derived miRNA spectra, supporting the concept of regulated RNA/miRNA trafficking from cellular compartments into the extracellular environment [45]. During coagulation, blood cells are subjected to a stress-inducing environment that can actively promote the release of specific miRNAs and other RNA species, a process that has also been observed in vitro under serum-free conditions [44].

Despite these biological and technical differences favoring serum, the findings of our meta-regression provide an important complementary perspective. Although pooled estimates showed that serum-derived miRNAs achieved the highest sensitivity, specificity, and AUC among the three matrices, the meta-regression demonstrated that the diagnostic performance of miRNA-based assays was not significantly influenced by the type of biofluid (saliva, serum, or plasma). The higher diagnostic odds ratio observed for serum was numerically superior but did not reach statistical significance (*p* > 0.65). This indicates that, once between-study variability and within-study assay structure are accounted for, the biological source of circulating miRNAs may be sufficiently consistent across oral and systemic biofluids. Accordingly, these results support the methodological flexibility of liquid biopsy approaches in OSCC, suggesting that clinically meaningful detection of tumor-associated miRNAs can be achieved across multiple biofluid types, even if serum tends to exhibit incremental empirical advantages in raw diagnostic metrics.

Our meta-analysis offers an important advantage over previous reviews, as no earlier study has directly compared diagnostic performance across all three major biofluid matrices: saliva, serum, and plasma within the same analytic framework. However, several limitations should be acknowledged. First, meta-regression was limited to biofluid type due to insufficient and heterogenous reporting of important covariates such as age, smoking and alcohol consumption, tumor stage, and miRNAs analytical methods across studies. As a result, residual confounding may exist, and the absence of a statistically significant matrix effect should be interpreted with caution, as the true influence of biofluid type may be underestimated. In addition, several included studies evaluated single-miRNA biomarkers, some of which are dysregulated across a broad range of non-malignant diseases, raising concerns regarding disease specificity. For example, miR-106a has been reported to be altered in inflammatory bowel disease, alzheimer’s disease, and multiple sclerosis, while miR-21 is a well-known inflammation and stress-responsive miRNA dysregulated in multiple cancers and non-malignant inflammatory conditions [48,49,50,51]. Consequently, diagnostic performance observed for single-miRNA markers may partly reflect non-specific inflammatory signals rather than OSCC-specific biology. In contrast, multi-miRNA panels may mitigate this limitation by capturing combinatorial expression patterns more representative of tumor-specific processes. Finally, these limitations point to key areas for improvement in future studies.

## 5. Conclusions

Among the three biofluids, serum-derived miRNAs showed the highest pooled performance, with superior sensitivity, specificity, and AUC. However, meta-regression indicated that biofluid type did not significantly influence overall diagnostic accuracy, suggesting that clinically relevant miRNA signatures can be reliably detected across different matrices.

## Figures and Tables

**Table 1:** PICOS (Population, Index test, Comparison, Outcomes, and Study design) criteria used for study selection.

Population (P)	Patients with histopathologically confirmed oral squamous cell carcinoma (OSCC)
Index Test (I)	Detection and quantification of miRNAs in non-invasive biofluids (saliva, plasma, or serum)
Comparison (C)	Healthy controls (no disease)
Outcomes (O)	Measures of diagnostic accuracy: sensitivity, specificity, area under the ROC curve (AUC), positive predictive value (PPV), negative predictive value (NPV), true positives (TP), false positives (FP), true negatives (TN), false negatives (FN)
Study Design (S)	Observational diagnostic accuracy studies (case–control, cohort)

**Table 2:** Characteristics of included studies.

First Author	Year	Country	Sample Size			Sample Type	miRNAs Evaluated	Expression Direction	miRNAs Type (Single/Panel)	Normalization/Internal Control	Detection Platform	
			OSCC	Control	Total						Discovery/ Profiling	Validation
Balakittnen et al. [19]	2024	Australia	50	60	110	Saliva	miR-7-5p, miR-10b-5p, miR-182-5p, miR-215-5p, miR-431-5p, miR-486-3p, miR-3614-5p, and miR-4707-3p	Up and Down	Panel	miR-191-5p, miR-484, and SNORD96A	NGS	RT-qpCR
Tarrad et al. [20]	2023	Egypt	12	12	24	Saliva	miRNA-106a	Down	Single	SNORD-68	_	RT-qPCR
Garg et al. [21]	2023	India	30	30	60	Saliva	miRNA-21	Up	Single	U6 snRNA	_	RT-qPCR
							miRNA-184	Down	Single		_	RT-qPCR
Scholtz et al. [22]	2022	Hungary	43	44	87	Saliva	miR-31-5p, miR-345-3p, and miR-424-3p	Up and Down	Panel	SNORD60	_	RT-qPCR
Mehterov et al. [23]	2022	Bulgaria	34	12	45	Saliva	miR-30c-5p	Down	Single	RNU6 and SNORD72	_	RT-qPCR
Romani et al. [24]	2021	Italy	50	42	92	Saliva	miR-16-5p, miR-484, and miR-191-5p	Up and Down	Panel	Arithmetic mean of three reference miRNAs: miR-16-5p, miR-484, and miR-191-5p	_	RT-qPCR
He et al. [25]	2020	China	49	14	63	Saliva	miR-24-3p	Up	Single	cel-miR-39	Microarray	RT-qpCR
Wan et al. [26]	2017	Australia	47	113	163	Saliva	miR-9, miR-127, miR-134, miR-191, miR-222, and miR-455	Up	Panel	SNORD96A	_	RT-qpCR
Duz et al. [27]	2016	Turkey	24	25	49	Saliva	miR-139-5p	Down	Single	RNU6B	Microarray	RT-qpCR
Zahran et al. [28]	2015	Saudi Arabia	20	20	40	Saliva	miR-21	Up	Single	SNORD68	_	RT-qpCR
							miR-145	Up	Single			
							miR-184	Up	Single			
Piao et al. [29]	2023	Korea	27	21	48	Serum	miR-92a-3p, miR-92b-3p, miR-320c, and miR-629-5p	Up	Panel	Combination of miR-16 and miR-423-5p	NGS	RT-qpCR
Mazumder et al. [30]	2023	India	47	42	89	Serum	miR-315p, miR-483-5p, let-7b-5p, miR-486-5p	Up	Panel	miR-16	_	RT-qPCR
Nakamura et al. [31]	2021	Japan	40	40	80	Serum	miR-24, miR-20a, miR-122, miR-150, miR-4419a, and miR-5100	Up and Down	Panel	miR-16	Microarray	RT-qPCR
Wang et al. [32]	2021	China	132	85	217	Serum	miR-206	Down	Single	U6 snRNA	_	RT-qPCR
Karimi et al. [33]	2020	Iran	20	20	40	Serum	miR-21	Up	Single	miR-191	_	RT-qpCR
							miR-24	Up	Single			
							miR-29a	Down	Single			
Chen et al. [34]	2018	China	121	55	176	Serum	miR-99a	Down	Single	cel-miR-39	_	RT-qPCR
Chang et al. [35]	2018	China	144	70	214	Plasma	miR-150-5p	Up	Single	Combination of miR-130b-3p and miR-221-3p	NGS	RT-qpCR
							miR-423-5p	Up	Single			
							miR-150-5p and miR-423-5p	Up	Panel			
Tachibana et al. [36]	2016	Japan	31	31	62	Plasma	miR-223	Up	Single	let-7a and RNU6B (U6)	Ultra-sensitive genome-wide miRNA array	RT-qPCR
Lu et al. [37]	2015	Taiwan	90	53	143	Plasma	miR-196a miR-196b	Up	Panel	NR	_	RT-qPCR
							miR-196a	Up	Single			
							miR-196b	Up	Single			

NGS Next Generation Sequencing, RT-qPCR Real Time Quantitative Polymerase Chain Reaction, NR Not Reported, SNORD Small Nucleolar RNA C/D Box, U6 snRNA U6 Small Nuclear RNA, RNU6B U6 Small Nuclear 2, let-7a Lethal-7a microRNA, Expression direction: “Up” denotes microRNAs upregulated in OSCC; “Down” denotes microRNAs downregulated compared with healthy controls.

**Table 3:** Baseline demographic and clinical characteristics of OSCC patients and control groups in the included studies.

First Author	Age		Gender				Smoking Habit				Systemic Disease Excluded	Control Definition	Matching Reported
	OSCC (Mean ± SD or Median [IQR])	Healthy Control (Mean)	OSCC		Control		OSCC		Control				
			Male (n, %)	Female (n, %)	Male (n, %)	Female (n, %)	Yes	No	Yes	No			
Balakittnen et al. [19]	64.8 (47–87)	67.4 (43–89)	38 (76)	12 (24)	38 (63.3)	22 (36.7)	7 (14)	31 (62)	34 (56.7)	26 (43.3)	NR	Clinically healthy individuals without OSCC or OPMD	NR
Tarrad et al. [20]	53.1 ± 8.0	38.7 ± 6.6	6 (50)	6 (50)	5 (41.7)	7 (58.3)	-	-	-	-	Yes	Systemically free individuals with no oral mucosal lesions, no systemic disease, no pregnancy/lactation, no current medication, and no clinical oral mucosal lesions on examination	Age, systemic disease
Garg et al. [21]	51.1 ± 12.75	38.5 ± 4.9	23 (76)	7 (24)	23 (76)	7 (24)	-	-	-	-	Yes	Age and gender-matched healthy control individuals who had no smoking habit and had no significant oral or systemic disease	Age, sex, smoking habit
Scholtz et al. [22]	57.9	57.6	28 (65)	15 (35)	16 (36)	28 (64)	24 (56)	12 (28)	6 (14)	21 (70)	NR	Individuals without diagnosis of OSCC	-
Mehterov et al. [23]	60.9 (48–72)	-	30 (88.2)	4 (11.98)	-	-	32 (94.1)	2 (5.9)	-	-	NR	Individuals with no oral mucosal lesions	NR
Romani et al. [24]	66.7 (30–90) * 64.75 (24–91) **	50.72 (22–92) * 75.57 (71–91) **	43 (70) * 19 (68) **	18 (30) * 9 (32) **	28 (64) * 10 (71) **	16 (36) * 4 (29) **	36 (59) * 13 (46) **	25 (41) * 14 (50) **	21 (48) * 5 (35) **	23 (52) * 4 (30) **	NR	Individuals with no oral lesions	Smoking habit
He et al. [25]	-	-	30 (61.2)	19 (38.8)	8 (57.1)	6 (42.9)	20 (40.8)	29 (59.2)	5 (35.7)	9 (64.3)	Yes	Individuals with no oral mucosal lesions and no other malignant tumors or severe systemic diseases	Age, sex, smoking habit, systemic disease
Wan et al. [26]	61.9 ± 11.1	44.7 ± 11.4	83 (82.2)	19 (17.8)	59 (52.2)	54 (47.8)	84 (83.2)	17 (16.8)	42 (37.3)	71 (63.7)	NR	Individuals with no previous history of any malignancies in the head and neck areas	NR
Duz et al. [27]	54.08 ± 2.38	46.88 ± 3.63	19 (76)	6 (24)	21 (84)	4 (16)	-	-	-	-	Yes	Age and gender-matched individuals without OSCC, without oral lesions and negative for hepatitis and HIV	Age, sex
Zahran et al. [28]	58 ± 9.2	51.1 ± 9.3	8 (40)	12 (60)	9 (45)	11 (55)	6 (30)	14 (70)	-	-	Yes	Individuals with no significant oral or systemic disease, and without periodontal disease	NR
Piao et al. [29]	65 ± 14.2	-	19 (70.4)	8 (29.6)	-	-	-	-	-	-	NR	Age and sex-matched healthy individuals with no diagnosis of OSCC	Age, sex
Mazumder et al. [30]	54.02 (30–79)	50.61 (20–64)	32 (68)	15 (31.9)	28 (66.7)	14 (33.3)	34 (72.3)	13 (27.7)	-	-	Yes	Individuals with no OPMD or OSCC, no specific systemic diseases reported	Age, sex, systemic disease
Nakamura et al. [31]	67.3	63.7	21 (52.5)	19 (47.5)	20 (50)	20 (50)	-	-	-	-	Yes	Individuals undergoing routine health screening with no pathognomonic signs and no diagnosis of OSCC	Age, sex
Wang et al. [32]	57.39 ± 19.28	-	87 (65.9)	45 (34)	-	-	81 (61.3)	51 (38.6)	-	-	NR	Individuals without oral cancer or other specified oral diseases	NR
Karimi et al. [33]	46.60 ± 10.69	47.10 ± 17.66	14 (70)	6 (30)	14 (70)	6 (30)	10 (50)	10 (50)	6 (30)	14 (70)	Yes	Individuals without history of malignancies, prior head and neck radio-/chemotherapy, immunodeficiency, and immune/autoimmune diseases	Age, sex, smoking habit
Chen et al. [34]	-	-	73 (60.3)	48 (39.7)	-	-	53 (43.8)	68 (56.2)	-	-	NR	Individuals with no diagnosis of OSCC	NR
Chang et al. [35]	52.20 ± 9.03 * 53.79 ± 11.25 **	52.05 ± 12.78 * 52.86 ± 14.06 **	31 (96.8) * 80 (97.5) **	1 (3.2) * 2 (2.5) **	20 (100) * 48 (96.0) **	0 (0) * 2 (4.0) **	31 (96.8) * 75 (91.5) **	1 (3.2) * 7 (8.5) **	13 (80) * 48 (96) **	4 (20) * 2 (4.0) **	NR	Individuals without clinical OL or OSCC (no oral potentially malignant disorder or carcinoma)	NR
Tachibana et al. [36]	75.74 ± 8.96	-	20 (64.5)	11 (35.4)	31 (100)	-					NR	Age and sex-matched individuals without oral cancer, recruited from the same hospitals	Age, sex
Lu et al. [37]	54.0 ± 11.7	47.2 ± 11.8	82 (91)	8 (9)	37 (70)	16 (30)	55 (61)	-	-	-	NR	Individuals without oral cancer or pre-cancer lesions	NR

OSCC oral squamous cell carcinoma; OPMD oral potentially malignant disorder; SD standard deviation; IQR interquartile range; NR not reported; n number of subjects; OL oral leukoplakia; HIV human immunodeficiency virus. * indicates the training set; ** indicates the validation set.

**Table 4:** Summary of diagnostic performance metrics of the included studies.

First Author	TP	FP	TN	FN	Sensitivity (%)	Specificity (%)	PPV (%)	NPV (%)	AUC
Balakittnen et al. [19]	43	7	6	54	86	90	87.8	88.5	0.95
Tarrad et al. [20]	12	3	0	9	100	70.8	63.2	100	0.90
Garg et al. [21]	24	9	21	6	80	70	72	79	0.89
Garg et al. [21]	24	8	22	6	80	74	75	79	0.87
Scholtz et al. [22]	37	10	34	6	86	77	78.7	85	0.87
Mehterov et al. [23]	43	11	31	7	86	74	79.6	81.6	0.82
Romani et al. [24]	24	2	12	4	85.4	85.1	92.3	75	0.92
He et al. [25]	32	3	11	17	64.4	80	91	39	0.74
Wan et al. [26]	28	7	106	19	60	94	80	85	0.82
Duz et al. [27]	18	4	21	7	75	84	82	75	0.81
Zahran et al. ^(a)^ [28]	65	7	35	13	65	65	90	73	0.73
Zahran et al. ^(b)^ [28]	60	6	40	14	60	70	91	74	0.68
Zahran et al. ^(c)^ [28]	80	5	20	15	80	75	94	57	0.86
Piao et al. [29]	17	0	15	6	97.8	73.9	100	71	0.90
Mazumder et al. [30]	14	4	8	3	80	64.3	77.8	72.7	0.72
Nakamura et al. [31]	22	3	37	18	55	92.5	88	67.3	0.84
Wang et al. [32]	107	23	62	25	81.2	72.7	82.3	71.3	0.85
Karimi et al. ^(a)^ [33]	19	1	19	1	95	95	95	95	0.95
Karimi et al. ^(b)^ [33]	16	6	14	6	80	70	73	70	0.75
Karimi et al. ^(c)^ [33]	20	0	20	0	100	100	100	100	1.00
Chen et al. [34]	97	9	46	24	80.2	83.6	92	66	0.91
Chang et al. ^(a)^ [35]	69	16	54	45	61	77	81	55	0.7
Chang et al. ^(b)^ [35]	67	19	51	47	59	73	70	61	0.68
Chang et al. ^(c)^ [35]	82	29	51	33	71	73	74	61	0.75
Tachibana et al. [36]	21	19	12	10	67.7	61.3	64	66	0.70
Lu et al. ^(a^) [37]	79	4	49	11	87.8	92.5	95	92.5	0.96
Lu et al. ^(b)^ [37]	60	2	50	30	67	96	97	63	0.85
Lu et al. ^(c)^ [37]	88	10	43	2	98	81	90	96	0.96

TP: True positive; FP: False positive; FN: False negative; TN: True negative; PPV: Positive Predictive Value; NPV: Negative Predictive Value; AUC: Area Under Curve. Supscripts (a), (b), and (c) indicate distinct microRNA targets or assay panels evaluated within the same study population.

**Table 5:** Comparison of diagnostic performance across saliva, serum, and plasma matrices using meta-regression of log diagnostic odds ratios (lnDOR).

Biofluid	Coefficient (lnDOR)	Exp(b) (DOR Ratio)	95% CI (exp)	*p*-Value	Interpretation
Saliva (reference)	–	–	–	–	Baseline category
Serum	0.3277	1.38	0.33–5.80	0.653	No significant difference vs saliva
Plasma	0.1256	1.13	0.35–3.72	0.836	No significant difference vs saliva
Intercept (_cons)	2.4538	11.63	4.57–29.64	<0.001	Pooled DOR (saliva reference)

## Data Availability

The original contributions presented in this study are included in the article and Supplementary Materials. Further inquiries can be directed to the corresponding author.

## Supplementary Materials

The following supporting information can be downloaded at: https://www.mdpi.com/article/10.3390/jpm16010052/s1, Table S1: PRISMA checklist.

## Abbreviations

The following abbreviations are used in this manuscript:

OSCC	Oral Squamous Cell Carcinoma
NGS	Next Generation Sequencing
RT-qPCR	Real-Time Quantitative Polymerase Chain Reaction
TP	True positive
FP	False positive
FN	False negative
TN	True negative
PPV	Positive Predictive Value
NPV	Negative Predictive Value
lnDOR	log Diagnostic Odds Ratios
CI	Confidence Interval
miR	miRNAs/microRNAs
PRISMA	Preferred Reporting Items for Systematic Reviews and Meta-Analyses
QUADAS-2	Quality Assessment of Diagnostic Accuracy Studies-2
HSROC	Hierarchical Summary Receiver Operating Characteristic
